# Caregivers’ perceptions of routine immunisation service performance across availability, accessibility, quality, responsiveness and satisfaction in Adamawa State, Nigeria: A mixed-methods study

**DOI:** 10.4102/phcfm.v18i1.5613

**Published:** 2026-10-21

**Authors:** Akolade U. Jimoh, Rauf I. Rauf, Sunday Atobatele, Daniel Azuka, Stephen O. Asaolu, Edikan Uwatt, Oladayo Awoyale, Akudo Alli, Ejovi Akpojaro, Temitope Kassim, Kehinde Olu-Onifade, Sidney Sampson

**Affiliations:** 1 Sydani Initiative for International Development, Sydani Group, Abuja, Nigeria; 2 Sydani Institute for Research and Innovation, Sydani Group, Abuja, Nigeria; 3 Department of Statistics, Faculty of Physical Sciences, University of Abuja, Abuja, Nigeria; 4 Sydani Group, Abuja, Nigeria

**Keywords:** community scorecard, immunisation caregiver experience, quality of care, vaccine uptake, mixed-methods research, Nigeria

## Abstract

**Background:**

Globally, 14.3 million children remain unvaccinated, with a large proportion residing in low- and middle-income countries, including Nigeria. Despite ongoing efforts to improve immunisation coverage, substantial gaps in uptake persist.

**Aim:**

This study assessed caregivers’ perceptions of routine immunisation service performance across five domains: availability, accessibility, quality of care, responsiveness and satisfaction in Adamawa State, Nigeria.

**Setting:**

Ten Local Government Areas in Adamawa State, Nigeria.

**Methods:**

A mixed-methods study combining a descriptive cross-sectional survey with qualitative in-depth interviews was conducted among caregivers of children under 5 years in 10 purposively selected local government areas (LGAs) in Adamawa State. Quantitatively, 685 caregivers were selected using probability proportional to size sampling from catchment settlements of supported facilities. Qualitatively, 120 caregivers (12 per LGA) were purposively selected for in-depth interviews. Data were collected using a community scorecard and a structured interview guide.

**Results:**

Routine immunisation performance varied across LGAs and domains. Guyuk recorded the highest scores for availability (81.3), quality of care (84.6), responsiveness (81.3), and satisfaction (89.2), while Toungo recorded the lowest scores across most domains. Accessibility demonstrated the greatest variability, with lower scores observed in Fufore, Numan and Toungo. Qualitative findings showed that respectful communication, perceived vaccine benefits and positive provider interactions enhanced caregiver satisfaction, while delays, travel burden and poor queue management reduced service experience.

**Conclusion:**

Improving session timeliness, health worker availability, vaccine supply and respectful communication may strengthen routine immunisation uptake and equity, particularly in lower-performing LGAs.

**Contribution:**

This study provides community-level evidence on routine immunisation service experience and satisfaction.

## Introduction

Vaccination, being an integral part of the primary health care system, is among the most cost-effective and efficient ways to prevent infectious diseases and their complications.^1,2,3^ It saves approximately 50 million lives and promotes optimal health and well-being for children and adults.^4^ Although vaccines offer incalculable benefits and immunisation rates have risen considerably across the globe over time, diseases that are preventable through vaccination still represent a leading contributor to childhood sickness and death within low- and middle-income countries.^5,6^ Worldwide, 14.3 million children received no vaccinations, known as zero-dose children, and 5.6 million were partially vaccinated.^7^ The bulk of these children live in low- and middle-income nations, where five African countries (Nigeria, Ethiopia, the Democratic Republic of Congo [DRC], Sudan and Guinea) jointly represent the region’s highest number of children who have either missed scheduled vaccinations or received no vaccines at all.^8^ Out of this, Nigeria accounts for a substantial share of the missed dose children.^7,9^ A key finding from Nigeria’s Demographic and Health Survey revealed that 31% of children aged 12–23 months have not received any vaccinations. The northeast region, more especially Adamawa State, contributes significantly to this burden.^10^ Strategies to address low immunisation coverage have been implemented in the past.^11^ Despite this, Nigeria continues to face challenges in attaining equitable and sustained immunisation coverage.^7^ Evidence indicates systemic problems, including inconsistent service delivery, missed opportunities and contextual barriers, including insecurity, mobility issues and health system constraints.^12^ Suboptimal quality of immunisation sessions and caregiver dissatisfaction have been reported in a previous study.^13^ Caregivers of unvaccinated and partially vaccinated children encountered a complex mix of overlapping obstacles, including gender dynamics, economic hardship, physical inaccessibility to health facilities, poor quality of service interactions^14^ and the limited availability of healthcare professionals.^15^ Similarly, caregivers most often face barriers, such as limited financial resources and a lack of information, when accessing vaccines. The study also noted that vaccine availability is impacted by supply chain issues and unpredictable demand. As for quality concerns, caregivers primarily worry about substandard vaccines, counterfeit combination vaccines, improper storage conditions and issues with manufacturing oversight.^16^ The quality of routine immunisation services was perceived as satisfactory by caregivers in an urbanised village.^17^ However, a population-based study found that only 20% of respondents were satisfied with immunisation services, with most expressing dissatisfaction.^18^ Previous research in Nigeria found that only 19.4% of caregivers were satisfied with immunisation services,^19^ and reports of poor service quality have also been documented.^20^ Challenges such as limited access to health facilities, caregivers’ time constraints, lack of sufficient knowledge about vaccines, shortages of vaccines or staff at healthcare facilities, missed vaccination opportunities, fears of minor side effects, limited access to vaccination services and caregivers’ beliefs about vaccines and poor quality were significant barriers to immunisation by caregivers.^18,21,22,23,24^ There is limited focus on comprehensive, multidimensional evaluations of service performance from users’ perspectives, specifically examining the availability, accessibility, acceptability, quality and satisfaction in routine immunisation. Using the community scorecard and Immunisation Caregiver Journey Interview (ICJI) framework to understand caregivers’ perspectives on vaccine uptake across domains in Adamawa State is relatively novel in this setting and can help contextualise the problem facing childhood immunisation in Adamawa State.^21,25,26,27,28^ This is crucial because child immunisation in Nigeria faces challenges related to logistical issues, systemic healthcare gaps and socio-economic factors that affect these domains.^29^ Employing a mixed-methods approach will provide a robust framework for understanding both the extent and the underlying reasons behind caregivers’ perceptions of immunisation services. In the study area, zero-dose immunisation remains a significant challenge,^10^ emphasising the need for a comprehensive evaluation of the problem through the lens of availability, accessibility, acceptability, quality and satisfaction among caregivers. Therefore, this study aims to assess caregivers’ perceptions of routine immunisation service performance across domains, including availability, accessibility, quality, responsiveness and satisfaction, in Adamawa State, Nigeria, using a mixed-methods design. By offering detailed evidence on measurable outcomes and lived experiences, the study aims to inform strategies to improve immunisation services and promote caregiver-centred care in similar resource-limited settings.

## Research methods and design

### Study design

This study employed a mixed-methods design combining a descriptive cross-sectional survey with qualitative in-depth interviews (IDIs) among caregivers of children under 5 years who accessed routine immunisation services in selected local government areas (LGAs) of Adamawa State, Nigeria. The mixed-methods approach enabled both quantitative assessment and contextual exploration of caregiver experiences with routine immunisation services. Quantitative findings provided measurable indicators of service performance, while qualitative inquiry generated deeper insights into caregivers’ perceptions and lived experiences across key dimensions of immunisation service delivery.

### Study setting

The study was conducted in 10 (Strengthening Routine Immunisation in Consequential Areas of Adamawa and Niger states) STRICAAN-supported LGAs in Adamawa State. They are Maiha and Mubi North LGAs in Adamawa North Senatorial District; Fufore, Girei and Gombi LGAs in Adamawa Central Senatorial District and Guyuk, Mayo-Belwa, Numan, Ganye and Toungo LGAs in Adamawa South Senatorial District. The 10 prioritised LGAs in the state were selected based on a combination of high zero-dose burden and the presence of ongoing support by other partners. One health facility was selected from each ward across the participating LGAs, resulting in a total of 104 health facilities included in the study. Settlements located within the catchment areas of the selected facilities were included to ensure representation of communities accessing routine immunisation services. Routine immunisation performance was assessed across five predefined domains: availability, accessibility, quality of care, responsiveness and overall caregiver satisfaction. The qualitative component complemented the quantitative assessment by providing a nuanced understanding of caregiver perceptions and experiences within these domains. Integration of both components enhanced methodological complementarity, triangulation and validity of the findings.

### Study population

The study population comprised caregivers of children under 5 years of age residing within the catchment communities of selected STRICAAN-supported health facilities across the ten participating LGAs out of 21 total LGAs in Adamawa State. Eligible participants included biological parents, guardians or other primary caregivers who were responsible for making healthcare decisions for the child and who had accessed routine immunisation services at the selected health facilities within the study period. For the qualitative component, the study population included caregivers with varied experiences of routine immunisation services to ensure diversity in perspectives across the assessed domains of availability, accessibility, quality of care, responsiveness and satisfaction.

#### Inclusion criteria

Participants were eligible for inclusion if they:

- Were aged 18 years or older.
- Were primary caregivers of at least one child under 5 years of age.
- Resided within the catchment area of the selected health facility.
- Had accessed routine immunisation services at the selected facility within the past 2 weeks.
- Were willing and able to provide informed consent.

#### Exclusion criteria

Participants were excluded if they:

- Had not utilised routine immunisation services at the selected health facility.
- Were unable to participate in the interview due to severe illness or communication difficulties.
- Declined to provide informed consent.

### Sample size and sampling procedure

The sample size for the quantitative component was calculated using Cochran’s formula for single population proportions (Equation 1)^30^:


$$n=\frac{z^{2}pq}{e^{2}}$$ [Eqn 1]

Assuming a 50% estimated proportion to maximise variability, a 95% confidence level and a 5% margin of error.^30^ After adjusting for a 10% non-response rate and applying a design effect of 1.5, the minimum required sample size was estimated at 640 caregivers. To improve statistical precision and generalisability, a total of 685 caregivers were recruited. A multistage sampling approach was employed. Initially, LGAs were purposively selected based on the following predefined epidemiological and programmatic criteria: LGAs with low Pentavalent-1 (Penta 1) coverage were prioritised to support identification of zero-dose children, while LGAs with recent or ongoing measles outbreaks were included because Measles-2 coverage served as a proxy indicator for full immunisation performance. Within selected LGAs, caregivers were recruited from settlements linked to one STRICAAN-supported health facility per ward. Settlements were selected using probability proportional to size (PPS) sampling. Eligible caregivers were subsequently recruited consecutively within selected settlements until the required sample size for each LGA was achieved. For the qualitative component, 12 caregivers were purposively selected from each LGA based on their experiences with routine immunisation services, resulting in a total of 120 in-depth interviews conducted across the ten LGAs.

### Data collection tools and procedure

Quantitative data were collected using a Community Score Card (CSC), a participatory governance and service-assessment tool adapted from the CARE Malawi CSC model.^26^ The CSC was used to assess caregiver perceptions of routine immunisation service performance across the five study domains. Qualitative data were collected using an interview guide adapted from the Immunisation Caregiver Journey Interview (ICJI) framework developed by Jalloh et al.^27^ The guide explored caregiver perceptions, experiences, barriers, facilitators and interactions with routine immunisation services. Data collection was conducted by trained research assistants with prior field experience, proficiency in Hausa and relevant local dialects and familiarity with primary health care settings. A two-day training workshop was organised for research assistants and field supervisors covering study objectives, ethical procedures, administration of study tools, interviewing techniques, interpersonal communication and practical role-play exercises to ensure standardisation and field preparedness. The study tools were subsequently piloted in a comparable non-study LGA to refine wording, improve clarity and ensure contextual appropriateness. Structured face-to-face interviews were conducted using handheld mobile devices programmed with Open Data Kit (ODK) forms. The electronic forms incorporated automated validation checks, including skip logic, range restrictions and mandatory-response fields, to minimise data-entry errors and improve data quality. Qualitative interviews were conducted by trained research team members in private and conducive settings, audio-recorded with participant consent and securely stored as coded audio files on a password-protected server. Each interview lasted between 35 min and 45 min. Daily field debriefing sessions were conducted to review data completeness, monitor field progress, address operational challenges and ensure adherence to sampling targets. Data collection was conducted over a 2-week period across the ten participating LGAs.

### Data processing and analysis

Quantitative data were exported from the ODK platform and analysed using RStudio version 4.5.2. Prior to analysis, datasets underwent systematic cleaning procedures including range validation, consistency checks, identification of outliers and management of missing data. Descriptive statistics were used to summarise participant characteristics and domain-specific responses. Likert-scale responses were transformed from a 1–5 scale into a standardised 0–100 scale to improve interpretability and facilitate comparisons across domains. Composite scores for availability, accessibility, quality of care, responsiveness and satisfaction were generated using domain-specific indicators. Individual indicator scores were aggregated to derive domain-level performance scores for each LGA, with higher scores indicating better perceived performance. Qualitative interviews were audio-recorded, transcribed verbatim, translated, validated and analysed using ATLAS.ti version 25 for Windows. Data analysis followed Braun and Clarke’s^31^ thematic analysis framework, involving familiarisation with transcripts, code generation, theme development, theme refinement and interpretation. Coding reliability was strengthened through intercoder review and consensus discussions among members of the research team. Emergent themes were subsequently mapped onto the Community Score Card domains to facilitate integration and triangulation with quantitative findings.

### Ethical considerations

Ethical clearance to conduct this study was obtained from the Adamawa State Government Ministry of Health and Human Services (No. S/MoH/1131/I). Ethical approval and institutional authorisation for the study were obtained from the Health Research Ethics Committee of Adamawa State Ministry of Health and Human Services with protocol number ADHREC 27/01/2026/23 and from the relevant ethical review and oversight bodies in Adamawa State. Written informed consent was obtained from all participants prior to data collection. Participants were assured of confidentiality, voluntary participation and the right to withdraw at any point without consequence. Individuals who are unable to provide informed consent in accordance with the principles of the Declaration of Helsinki were excluded from the study.

## Results

### Sociodemographic characteristics of the caregivers and the children

The sociodemographic characteristics of caregivers and their children receiving routine immunisation are presented in Table 1. The study population comprised predominantly female caregivers (93.4%), with the majority being mothers of the child (92.7%). Most respondents were married or cohabiting (88.6%). In terms of education, the largest proportion had at least secondary education (56.7%), while smaller proportions had no formal education (15.8%), primary education (14.2%) or Qur’anic or non-formal education only (13.4%). Respondents were largely within the reproductive age group, with a mean age of 30.5 years. The highest proportions were aged 25–29 years (30.4%) and 30–34 years (24.8%). Most households had one (53.1%) or two (36.2%) children under 5 years. Child characteristics showed a near-balanced sex distribution (53.3% male and 46.7% female). The mean age of children was 14.1 months, with the largest proportions in the 6–11 months (25.9%) and 12–23 months (23.4%) age groups. Additionally, 44.5% of caregivers reported that the index child was their first child receiving routine immunisation.

**TABLE 1:** Sociodemographic characteristics of caregivers and their children (*N* = 685).

Characteristic	*n*	%
**A. Caregiver characteristics**		
**Respondent age group (years)**		
16–19	19	2.8
20–24	135	19.7
25–29	208	30.4
30–34	170	24.8
35–39	97	14.2
40–44	32	4.7
45–49	13	1.9
50–54	8	1.2
55+	3	0.4
**Sex of respondent**		
Female	640	93.4
Male	45	6.6
**Marital status**		
Single	26	3.8
Married or cohabiting	607	88.6
Divorced or separated	36	5.3
Widowed	16	2.3
**Highest level of education**		
No formal education	108	15.8
Qur’anic or non-formal only	92	13.4
Primary	97	14.2
Secondary	284	41.5
Tertiary	102	14.9
Other	2	0.3
**Main occupation**		
Housewife or home duties	181	26.4
Farmer	227	33.1
Trader or business	200	29.2
Civil servant	36	5.3
Artisan	1	0.1
Student	6	0.9
Unemployed	32	4.7
Other	2	0.3
**Relationship to child**		
Mother	635	92.7
Father	24	3.5
Grandmother	14	2.0
Grandfather	2	0.3
Aunt or uncle	9	1.3
Other	1	0.1
**Number of children < 5**		
1	364	53.1
2	248	36.2
3	56	8.2
4+	17	2.5
**B. Child characteristics**		
**Child age group (months)**		
0–5	204	29.8
6–11	177	25.8
12–23	160	23.4
24–35	54	7.9
36–47	31	4.5
48+	59	8.6
**Sex of child**		
Male	365	53.3
Female	320	46.7
**First child receiving RI**		
Yes	305	44.5
No	380	55.5

*n*, Frequency; %, Percentage; RI, routine immunisation.

### Scorecard for availability, accessibility, quality-of-care, responsiveness and satisfaction of routine immunisation services

The composite domain scores for availability, accessibility, quality-of-care, responsiveness and satisfaction of routine immunisation services are shown in Table 2.

**TABLE 2:** Availability, accessibility, quality-of-care, responsiveness and satisfaction of routine immunisation services across study local government areas in Adamawa State (0–100 scale).

LGAs	Domain scores				
	Availability	Accessibility	Quality-of-care	Responsiveness	Satisfaction
Fufore	70.1	66.6	75.4	67.0	68.0
Ganye	77.0	76.5	79.0	79.8	84.3
Girei	73.7	75.1	77.4	74.8	76.5
Gombi	79.4	73.1	80.1	76.0	80.3
Guyuk	81.3	68.3	84.6	81.3	89.2
Maiha	78.2	72.1	75.8	71.2	82.2
Mayo-Belwa	77.2	74.6	79.3	76.6	79.3
Mubi North	76.2	77.4	77.5	76.0	82.4
Numan	75.0	66.2	73.8	71.6	82.2
Toungo	51.3	45.9	62.3	50.1	63.1

LGA, local government area.

### Availability

Availability of routine immunisation services varied across LGAs, but overall performance was relatively strong, with most LGAs recording availability domain scores above 70% (Table 2). The highest performance was observed in Guyuk (81.3%), followed by Gombi (79.4%) and Maiha (78.2%). Other LGAs, including Ganye (77.0%), Mayo-Belwa (77.2%) and Mubi North (76.2%), also demonstrated stable performance with most item scores above 75%. In contrast, moderate performance was observed in Numan (75.0%) and Girei (73.7%), while Fufore (70.1%) showed lower scores driven by gaps in session timeliness (68.0%) and avoidance of postponements (62.2%) (Table 3). Toungo recorded the lowest performance (51.3%), with markedly low scores in session timeliness (24.3%), session-day information availability (36.4%) and service postponement (50.0%) (Table 3). Despite generally high structural readiness, reported service disruptions reveal important operational challenges (Figure 1). Long delays before service initiation were the most frequently reported issue, particularly in Toungo (85.7%), Fufore (62.8%) and Numan (54.1%). Vaccine stock-outs were relatively low in high-performing LGAs such as Gombi (5.3%) and Maiha (8.9%) but were more pronounced in Toungo (37.1%) and Girei (31.7%). Health worker unavailability was most prominent in Fufore (46.5%), while remaining minimal in LGAs such as Numan (0.0%) and Mubi North (1.3%). Session cancellations or postponements were generally uncommon but were higher in Fufore (25.6%), Girei (24.4%) and Toungo (22.9%).

**FIGURE 1:**
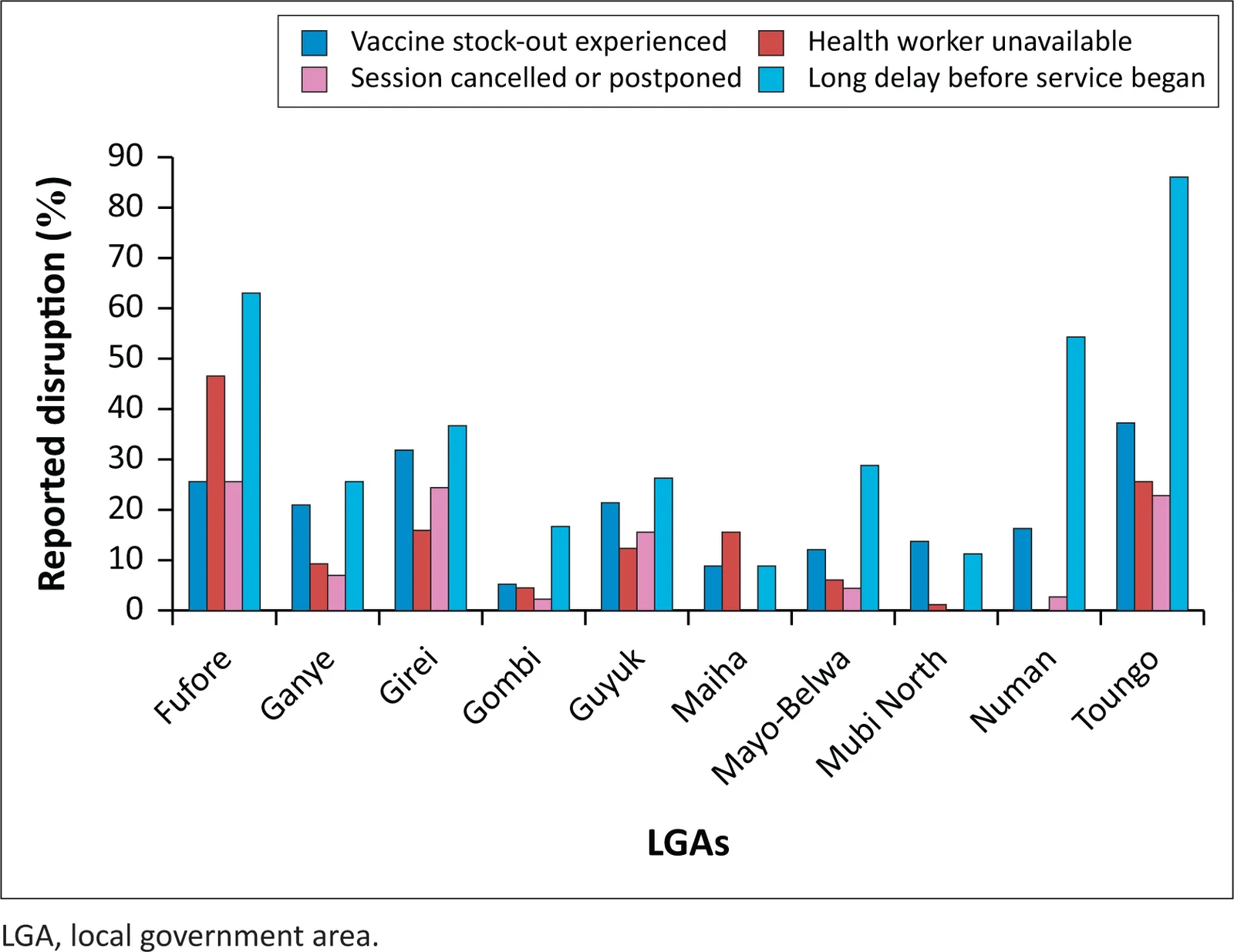
Reported Immunisation service disruptions in study local government areas.

**TABLE 3:** Standardised availability item scores by local government area (0–100 scale).

LGA	Services available on visit day	Needed vaccine available	Health worker present	Materials or supplies ready	Session started on time	No unnecessary postponement	Session-day information available	Session reliable when due
Fufore	74.4	75.6	72.1	75.0	68.0	62.2	70.9	62.2
Ganye	76.7	77.3	77.9	75.0	75.6	76.7	77.9	79.1
Girei	69.5	67.1	76.2	74.1	74.1	72.9	77.1	78.4
Gombi	80.7	81.3	80.1	80.3	75.2	78.6	78.6	80.3
Guyuk	78.7	82.8	89.8	78.3	76.8	75.8	85.0	83.6
Maiha	79.4	81.7	81.7	80.6	75.6	78.3	73.6	74.4
Mayo-Belwa	78.4	76.1	82.2	77.7	74.6	77.8	75.0	75.8
Mubi North	81.2	74.7	76.9	74.7	75.6	75.0	75.6	75.6
Numan	79.1	78.4	77.7	75.7	66.9	70.9	75.0	75.7
Toungo	59.3	51.4	67.9	64.3	24.3	50.0	36.4	57.1

LGA, local government area.

Qualitative findings from Fufore, Ganye and Girei LGAs confirmed that the availability of routine immunisation services was variable across settings. While some caregivers, particularly in Ganye, reported timely service delivery, the presence of health workers and clear communication through immunisation cards and community announcements, others experienced gaps in service readiness. In Girei and parts of Ganye, delays due to vaccine unavailability and late arrival of health workers affected session timing, while in Fufore, poor service organisation and queue management reduced perceived efficiency:

‘They immunize the children immediately when we go. There is no queue. We give them the card, they fill it, and vaccinate the child. Then we return home.’ (IDI, Participant 6, Ganye)

‘Sometimes if we go to the facility they will say let’s wait small because the person that will do the immunisation is on his way coming.’ (IDI, Participant 2, Ganye)

‘One time, I came with my child for vaccination, and they told me that they had gone to bring the vaccine. I waited for a while before they brought the vaccine.’ (IDI, Participant 4, Girei)

‘I do go there early in the morning. Instead of them to start with the first to come, they were attending to anyone who went to meet them … Things like these can be annoying. The only thing is to keep quiet.’ (IDI, Participant 11, Fufore)

### Accessibility

Accessibility of routine immunisation services showed greater variability across LGAs compared to availability, with domain scores ranging from 45.9% to 77.4% (Table 2). Overall, Mubi North recorded the highest accessibility score (77.4%), followed by Ganye (76.5%) and Girei (75.1%), reflecting relatively favourable conditions across key indicators such as ease of reaching services, affordability, convenience of schedules and acceptable waiting times. Other LGAs, including Mayo-Belwa (74.6%), Gombi (73.1%) and Maiha (72.1%), also demonstrated moderately high accessibility, although with some variation across specific components.

In contrast, Fufore (66.6%) and Numan (66.2%) recorded lower accessibility scores (Table 2), largely driven by challenges related to physical access, waiting time and cost. Guyuk (68.3%) (Table 2) also showed moderate performance, despite relatively high scores in awareness of service time and place (85.4%) and travel safety (80.9%) (Table 4). Toungo recorded the lowest accessibility score (45.9%) (Table 2), with consistently low performance across nearly all indicators, particularly ease of reaching services (41.4%), affordability (44.3%) and waiting time acceptability (30.0%) (Table 4). Supporting indicators further illustrate the nature of these accessibility challenges. Service access imposed opportunity costs on caregivers (Figure 2). Missed duties were common, particularly in Ganye (81.4%), Fufore (76.7%) and Guyuk (71.3%). In addition, waiting time emerged as a critical barrier, with mean waiting times highest in Guyuk (125.7 min), Toungo (85.3 min) and Fufore (71.1 min), compared to much shorter waiting times in Mubi North (22.8 min) and Ganye (24.0 min) (Figure 2). Correspondingly, the proportion of respondents reporting long or very long waiting times was highest in Toungo (54.3%), Numan (43.2%) and Guyuk (42.6%), reinforcing the burden of delays in these LGAs (Figure 2).

**FIGURE 2:**
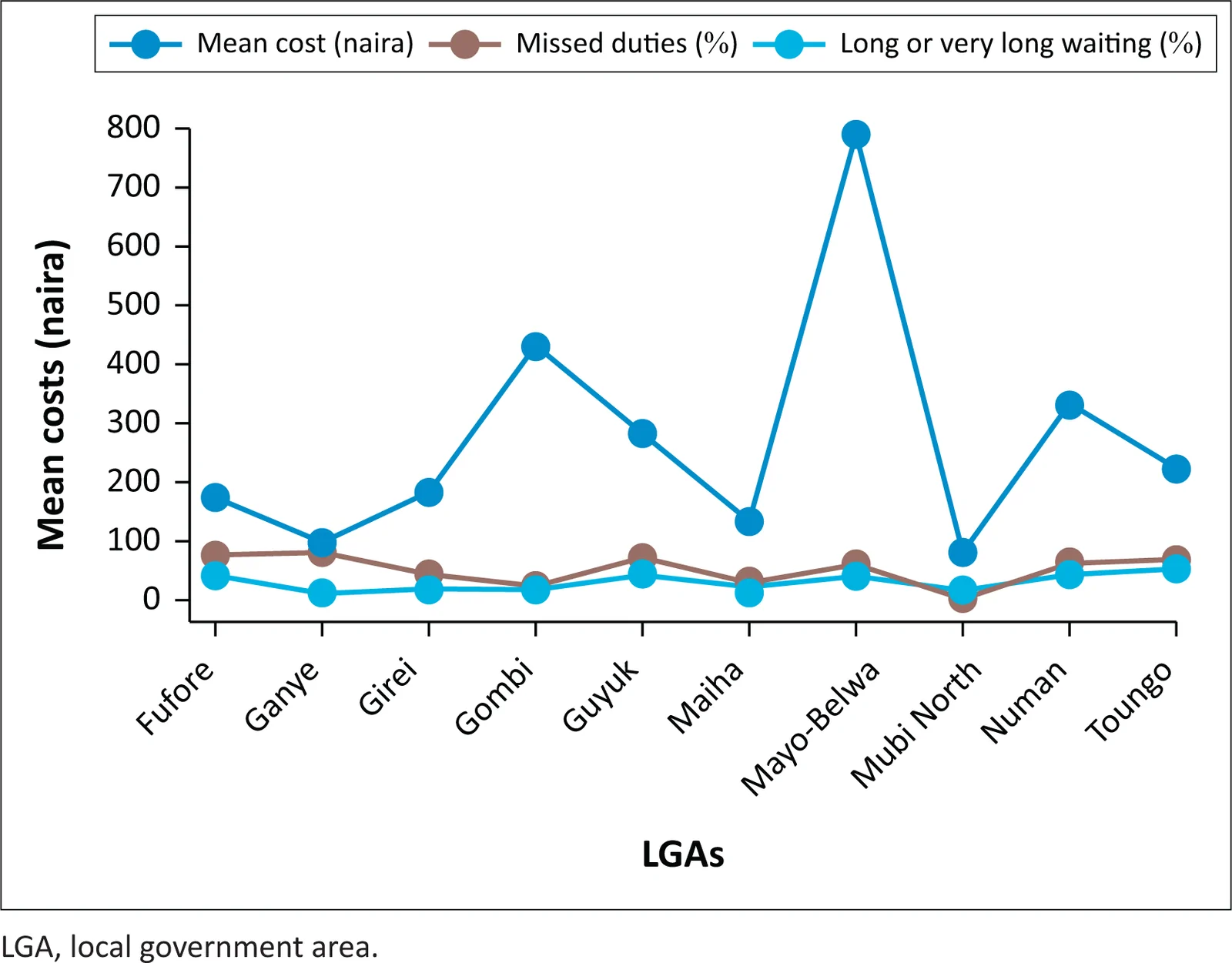
Variation in costs (₦), missed duties (%) and waiting time across local government areas (%).

**TABLE 4:** Standardised accessibility item scores by local government area (0–100 scale).

LGA	Easy to reach	Cost affordable	Schedule convenient	Easy to know time or place	Travel is safe	Location not discouraging	Waiting time acceptable
Fufore	58.1	70.3	69.8	68.6	65.7	70.3	63.4
Ganye	72.1	73.8	73.8	78.5	77.9	80.8	78.5
Girei	69.8	76.2	78.7	77.7	77.4	75.6	70.4
Gombi	70.6	68.4	74.1	75.6	74.8	73.5	74.8
Guyuk	56.1	54.7	69.5	85.4	80.9	70.7	60.5
Maiha	69.4	70.0	76.1	76.7	75.0	72.2	65.0
Mayo-Belwa	70.1	70.8	78.0	74.6	79.5	77.7	71.2
Mubi North	77.5	81.6	76.6	75.6	75.3	78.9	76.3
Numan	62.8	62.8	70.9	72.3	74.3	64.2	56.1
Toungo	41.4	44.3	51.4	51.4	56.4	46.4	30.0

LGA, local government area.

Qualitative findings across LGAs corroborated that accessibility to routine immunisation services was uneven. While some caregivers, particularly in Ganye, Girei and Maiha, reported facilities as close and easily reachable on foot, others in LGAs such as Gombi, Guyuk, Mubi North and Numan described longer, more difficult journeys requiring trekking, motorcycles or tricycles, often under challenging conditions. Transport mode and cost were central constraints, with caregivers frequently highlighting the financial burden of travel despite vaccines being free and linking these costs to delayed or missed visits. Caregivers were generally well informed about immunisation schedules through cards, verbal instructions and community announcements, which supported attendance. However, overall convenience was reduced by long travel times, service delays and extended waiting periods. Waiting time emerged as a consistent barrier across LGAs, with reports ranging from minimal delays to several hours due to crowding, staff shortages or late vaccine availability:

‘It takes me about 4 to 5 minutes to walk to the facility.’ (IDI, Participant 7, Ganye)

‘If it’s possible, the government should come to the community, and build a hospital … because we the people, we are suffering, with trekking.’ (IDI, Participant 2, Guyuk)

‘Before fuel was cheaper, it was ₦ 200 going, ₦200 naira coming, but now that fuel is expensive, but now is ₦250 going, and ₦250 coming.’ (IDI, Participant 3, Girei)

‘We stay very long when we go for vaccination because sometimes, we stay up till 3:00 pm. The first time I went with my child, I almost cried.’ (IDI, Participant 1, Gombi)

### Quality of care

Quality of care for routine immunisation services was generally high across LGAs, with most recording domain scores above 75% (Table 2). Guyuk had the highest quality-of-care score (84.6%), followed by Gombi (80.1%), Mayo-Belwa (79.3%) and Ganye (79.0%). Mubi North (77.5%) and Girei (77.4%) also demonstrated consistently good quality across most indicators. Moderate performance was observed in Maiha (75.8%), Fufore (75.4%) and Numan (73.8%), where some gaps were noted, particularly in communication-related indicators such as explaining vaccines or services and addressing caregivers’ questions. Toungo recorded the lowest quality-of-care score (62.3%), with notably low scores in explaining services (55.0%), explaining side effects (55.0%), answering questions (48.6%) and overall perception of quality (51.4%) (Table 5). Despite generally favourable quality scores, reported adverse experiences reveal important concerns in specific LGAs (Figure 3). Fufore recorded the highest proportion of caregivers reporting rude or disrespectful treatment (41.9%), followed by Gombi (15.2%), Toungo (11.4%) and Numan (10.8%). In contrast, such experiences were rare in LGAs like Mubi North (1.3%) and Guyuk (2.5%). Another adverse experience reported by the caregivers is that they were asked to pay for vaccination cards at some health facilities. This was relatively commonplace in Numan LGA and less common in Fufore, Toungo and Ganye LGAs.

**FIGURE 3:**
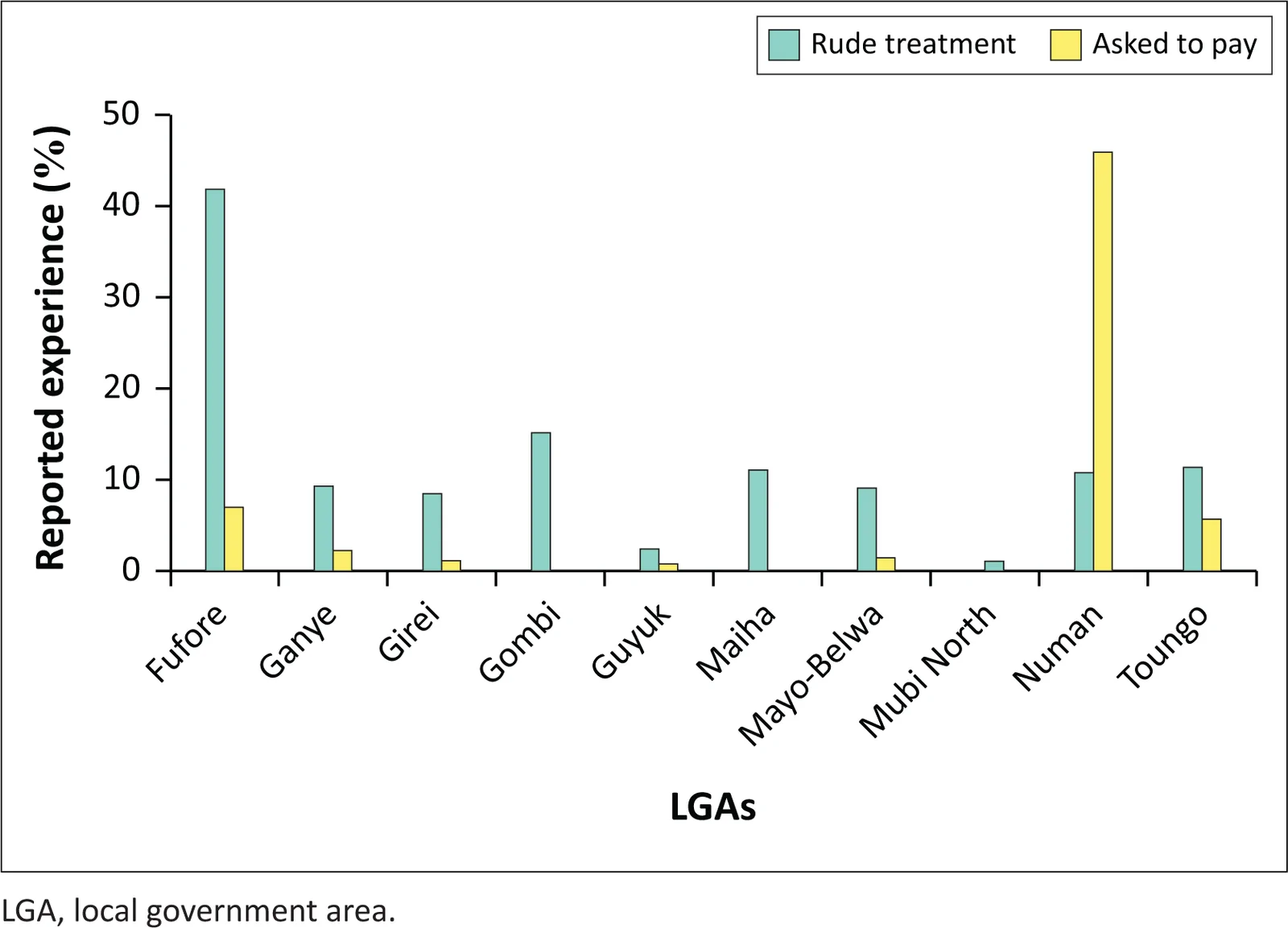
Reported adverse quality-of-care experiences by local government area.

**TABLE 5:** Standardised quality-of-care item scores by local government area (0–100 scale).

LGA	Respectful treatment	Handled child carefully	Explained vaccine or service	Explained side effects	Answered questions clearly	Checked card properly	Gave next visit date	Area clean and organised	Trusted competence	Comfortable discussing concerns	Process well organised	Thus, quality good
Fufore	64.0	75.0	72.7	77.9	72.1	79.1	81.4	80.2	77.9	72.1	75.6	76.2
Ganye	82.6	79.1	78.5	81.4	78.5	79.1	79.1	76.2	77.9	76.7	79.1	79.1
Girei	73.5	76.5	75.0	76.2	78.0	77.7	77.1	80.2	80.2	79.9	77.1	77.1
Gombi	72.5	80.1	82.0	79.5	79.9	79.5	82.0	81.4	79.9	79.3	82.0	83.3
Guyuk	82.0	85.2	83.2	82.8	84.4	87.7	86.7	85.0	87.7	84.8	81.1	85.0
Maiha	75.0	75.0	67.8	74.4	75.6	77.8	77.2	78.3	78.3	72.8	78.3	79.4
Mayo-Belwa	74.2	80.7	79.2	77.7	80.3	84.8	80.7	80.3	75.4	79.2	77.7	80.7
Mubi North	76.9	76.9	75.9	75.9	79.4	78.8	78.1	81.2	76.6	75.6	79.1	75.6
Numan	75.7	75.7	66.2	74.3	68.9	76.4	76.4	73.6	74.3	68.2	74.3	75.0
Toungo	60.0	72.1	55.0	55.0	48.6	80.0	69.3	69.3	75.0	53.6	58.6	51.4

LGA, local government area.

Qualitative findings across LGAs indicate that the perceived quality of routine immunisation services was generally positive but varied across settings. In LGAs such as Ganye, Girei, Mubi North and Numan, caregivers frequently described health workers as respectful, supportive and competent, with clear communication on vaccines, side effects and return dates. These elements, respectful treatment, effective communication and proper appointment management, were central to caregivers’ trust and continued use of services. However, quality was not consistent across all LGAs. In Fufore, caregivers reported poor attention, delays and inadequate explanation, while in Girei and Gombi, some accounts highlighted rudeness, staff shortages and weak service organisation. Explanation of side effects and communication of next visit dates were strong positive aspects across several LGAs and reinforced confidence in provider competence:

‘When a person takes his children to the hospital, they will not pay attention to him, someone might even get angry and leave; another person will decide not to go back there because of this.’ (IDI, Participant 6, Fufore)

‘They should explain things adequately: if there is a problem associated with the vaccine, bring your child back in the morning. They’re supposed to be telling us things like these.’ (IDI, Participant 11, Fufore)

‘When I arrived, I saw the workers seated and waiting for people to come so that they could immunize them. I saw a few people there, and I joined the queue. When they reached the queue, they attended to me cordially.’ (IDI, Participant 4, Girei)

‘I have taken my child three times, and I have never had any problem, honestly.’ (IDI, Participant 3, Mubi North)

### Responsiveness

Responsiveness and client experience varied across LGAs, with most recording moderate to high performance although notable disparities were observed (Table 2). Guyuk recorded the highest responsiveness domain score (81.3%), followed by Ganye (79.8%), Mayo-Belwa (76.6%) and both Gombi and Mubi North (76.0%), reflecting strong performance in listening to caregivers, responding to needs and creating a welcoming service environment. Girei (74.8%) also demonstrated relatively consistent performance across most indicators. Moderate performance was observed in Numan (71.6%), Maiha (71.2%) and Fufore (67.0%), where gaps were evident in timeliness of attention and respect for caregivers’ time. Toungo recorded the lowest responsiveness score (50.1%), with particularly low performance in timely attention (17.9%) and respect for caregivers’ time (20.7%) (Table 6). Across LGAs, indicators related to communication (e.g., listening to concerns and explaining processes) were generally stronger than those related to timeliness and efficiency.

**TABLE 6:** Standardised responsiveness and client experience item scores by local government area (0–100 scale).

LGA	Listened to concerns	Attended timely	Responded to needs	Patient with caregivers	Process easy to understand	Informed about reaction	Respected time	Felt welcomed	Comfortable returning	Would recommend point
Fufore	64.0	63.4	75.6	66.3	62.8	70.3	64.0	59.3	65.1	79.1
Ganye	79.7	78.5	81.4	81.4	81.4	79.1	76.7	79.7	79.7	80.8
Girei	76.8	71.0	76.8	78.0	76.2	74.7	68.3	76.2	75.3	75.0
Gombi	76.7	69.9	79.5	72.3	78.0	79.5	71.6	78.0	78.2	76.5
Guyuk	86.5	73.6	85.7	84.8	79.5	84.6	68.9	84.4	82.4	82.8
Maiha	72.2	61.1	75.0	68.3	73.9	77.8	65.0	66.7	76.7	75.0
Mayo-Belwa	74.2	71.6	79.2	78.0	76.1	76.9	76.9	79.2	78.0	75.8
Mubi North	76.2	74.7	75.6	75.9	75.6	78.1	75.3	75.3	76.6	76.6
Numan	66.2	64.9	74.3	70.3	74.3	75.0	58.8	76.4	78.4	77.0
Toungo	47.1	17.9	57.1	64.3	52.9	48.6	20.7	56.4	65.7	70.0

LGA, local government area.

Qualitative findings across LGAs indicate that responsiveness of routine immunisation services was present but also varied across settings. Caregivers in Fufore, Ganye, Girei, Maiha, Mayo-Belwa and Toungo commonly reported receiving reminders through health workers, immunisation cards, announcements and sometimes family members, which supported attendance and continuity of care. Many also described receiving explanations about vaccines and possible reactions, suggesting that service processes were generally understandable and supportive.

However, responsiveness was inconsistent. In Fufore, Ganye, Gombi, Guyuk, Mubi North and Numan, caregivers reported delays, long waiting times and occasional service interruptions, indicating that timely attention and service efficiency were not always assured:

‘They do give us date. If they say a month and some weeks, you have the immunisation card with you, once it’s time you can go there.’ (IDI, Participant 6, Fufore)

‘Even when we complained, they said they were sorry.’ (IDI, Participant 1, Toungo)

‘Yes, they tell us that when the vaccination site on the leg is swollen, we should come back to the hospital and they will put ice on it.’ (IDI, Participant 6, Ganye)

‘Sometimes if we go to the facility they will say let’s wait small because the person that will do the immunisation is on his way coming.’ (IDI, Participant 2, Ganye)

‘I do go there early in the morning. Instead of them to start with the first to come, they were attending to anyone who went to meet them … Things like these can be annoying. The only thing is to keep quiet.’ (IDI, Participant 11, Fufore)

### Overall satisfaction

Satisfaction with routine immunisation services was generally high across LGAs although notable differences were observed (Table 2). Most LGAs recorded satisfaction domain scores above 75%. Guyuk had the highest satisfaction score (89.2%), followed by Ganye (84.3%), Mubi North (82.4%) and both Maiha and Numan (82.2%), reflecting strong performance across overall satisfaction, increased confidence in services and willingness to return for future immunisation visits. Other LGAs, including Gombi (80.3%), Mayo-Belwa (79.3%) and Girei (76.5%), also demonstrated relatively high satisfaction although with slightly lower scores in specific components such as confidence in services. In contrast, Fufore (68.0%) and Toungo (63.1%) recorded the lowest satisfaction levels, driven by lower scores in overall satisfaction and confidence in routine immunisation services. For example, Toungo had particularly low scores for overall satisfaction (61.4%) and confidence (60.0%), indicating weaker caregiver experiences in this LGA. Across LGAs, willingness to return for the next immunisation visit was consistently high, exceeding 85% in most LGAs and reaching as high as 97.7% in Ganye and 97.6% in Guyuk (Figure 4).

**FIGURE 4:**
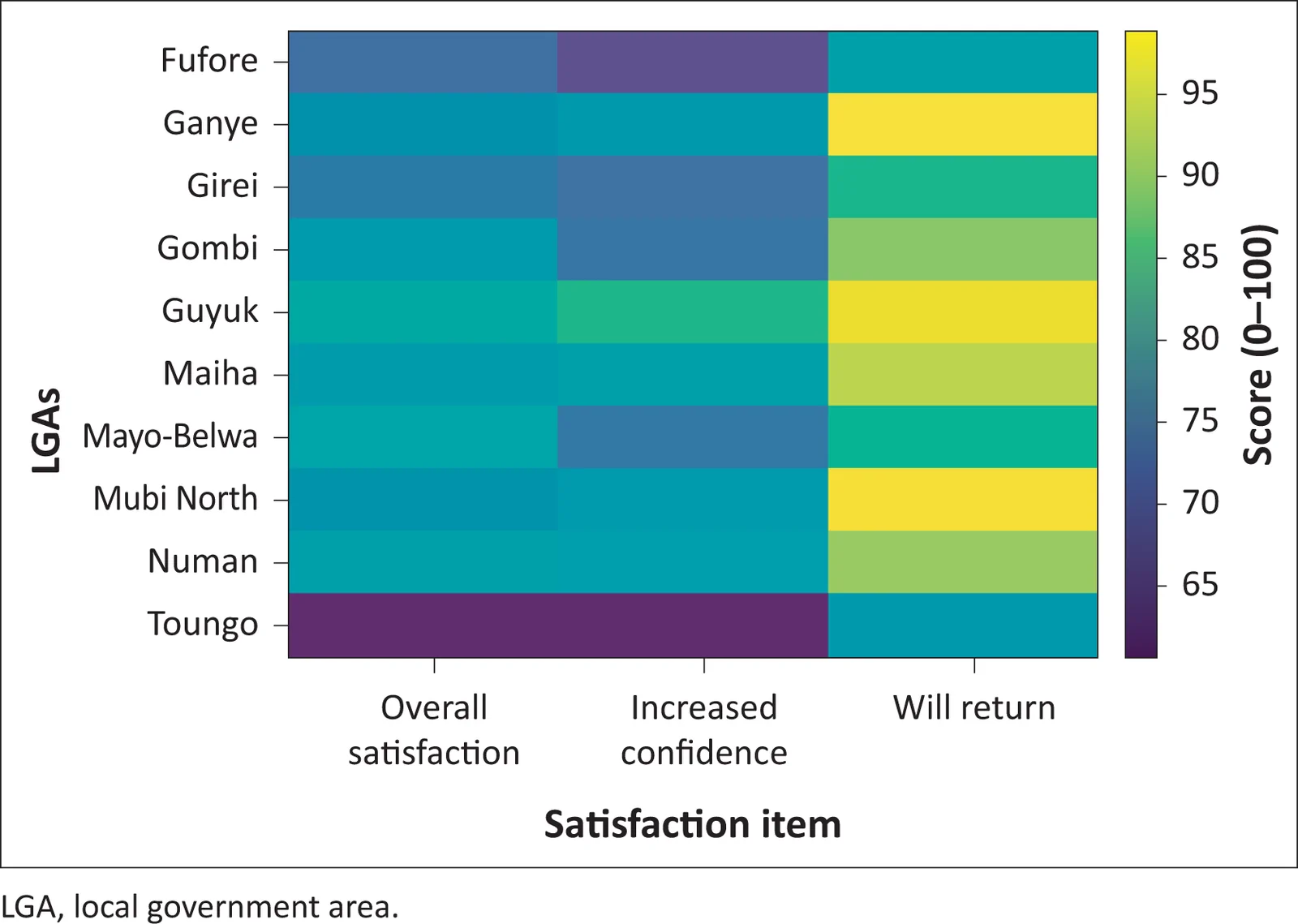
Standardised overall satisfaction item scores by local government area.

Qualitative findings across LGAs indicate that caregiver satisfaction with routine immunisation services was generally positive but varied across settings. Many caregivers expressed satisfaction and perceived vaccination as beneficial, linking positive experiences, such as respectful staff interactions, smooth service delivery and observed child health benefits, to increased confidence in routine immunisation and willingness to continue.

However, satisfaction was sometimes reduced by negative experiences, including poor attention, long waiting times, crowding, rude communication and service delays, particularly in LGAs such as Fufore and Gombi. Despite these issues, intention to return remained strong across LGAs, as caregivers continued to value immunisation for its role in protecting child health:

‘The experience was good because there were not many people there.’ (IDI, Participant 10, Girei)

‘The benefits I got, is a progress to my family, because I had no problem with my child, my child is growing in sound health.’ (IDI, Participant 4, Gombi)

‘I was satisfied because … she took her complete vaccine, and I also know she will not be affected by all these diseases that she got the vaccines for.’ (IDI, Participant 7, Mayo-Belwa)

‘Honestly, my mind is completely at ease because I know they are trying and they take good care of their patients.’ (IDI, Participant 7, Mubi North)

## Discussion

This study found that caregivers’ perceptions of routine immunisation services in Adamawa State were generally favourable, but service performance was uneven across LGAs. Quantitative findings show that most LGAs recorded relatively high domain scores for availability, quality, responsiveness and satisfaction although accessibility showed greater variation, and a few LGAs performed consistently worse than others. Toungo emerged as the weakest-performing LGA across nearly all domains, while Fufore also showed recurring weaknesses, particularly around timeliness, waiting time, interpersonal care and overall client experience. By contrast, LGAs such as Guyuk, Gombi, Ganye, Mubi North and Maiha tended to perform better across multiple domains. The qualitative findings help explain these patterns by showing that caregivers generally valued immunisation and remained committed to completing schedules, but their actual experience of services depended heavily on whether sessions were timely, accessible, respectful and predictable. The relatively strong availability scores across most LGAs suggest that routine immunisation services were present and operational in many settings. However, our findings indicate that availability extends beyond the mere existence of services to include their reliable and timely delivery. In lower-performing LGAs such as Toungo, Girei and Fufore, delays in session commencement, localised vaccine stock-outs and intermittent health worker unavailability point to gaps in operational readiness. Qualitative findings further support this interpretation, showing that although caregivers were often aware of scheduled immunisation sessions through cards, verbal instructions or announcements, they still encountered delays, postponed attention or late arrival of vaccinators at the service point. This suggests that the primary challenge in these settings is not the absence of services but inconsistencies in service delivery and continuity. These findings are consistent with evidence from other settings. In Pakistan, suboptimal immunisation service has been linked to inefficient service delivery processes, highlighting how systemic weaknesses can undermine the effective functioning of otherwise available services.^32^ Similarly, evidence from Guatemala indicates that barriers to immunisation are more strongly associated with challenges in accessing and obtaining optimal services, such as long waiting times and service inefficiencies.^33^ Further studies from Southwest Nigeria and Ethiopia have documented that vaccination may be deferred due to operational constraints, including the need to meet minimum thresholds before opening multi-dose vials.^34,35^ Such practices mean that children presenting for immunisation are not always vaccinated immediately, underscoring that effective availability depends on continuous operational readiness and the ability to deliver services at the point of need, rather than simply the presence of immunisation services.

Accessibility emerged as one of the clearest weak points in the service experience. Quantitative findings showed that accessibility varied more widely than availability, with Toungo performing worst and Ganye, Girei and Mubi North performing comparatively better. Travel time, transport cost and waiting time were all substantial burdens. Qualitative findings from this study deepen this by showing how these barriers were experienced in practice: some caregivers could walk to nearby facilities within minutes, while others had to trek long distances, cross difficult terrain or pay for motorcycles or tricycles when money was available. Even where vaccination itself was free, the cost of transport and time away from household responsibilities created real barriers to use. Consistent with our findings, evidence from multiple settings highlights that the presence of immunisation services does not necessarily translate into equitable access when practical barriers persist. A study from southern Rajasthan in India demonstrates that the hidden costs of vaccination, including lost wages, time spent and transportation expenses incurred due to long travel distances, can act as significant impediments to immunisation service utilisation.^36^ Similarly, evidence from Nigeria indicates that geographical and financial constraints, such as poor road infrastructure and long distances to health facilities, increase transportation costs and limit caregivers’ ability to access immunisation services.^37,38^ Time-related barriers further compound these challenges. For instance, in Cameroon, prolonged waiting times for vaccination, ranging from under 20 min in some regions to over 4 h in others, have been documented, reflecting inefficiencies at the point of service delivery.^39^ In Southwest Nigeria, caregivers also frequently cited time constraints as a reason for missing scheduled vaccinations.^38^ Together, this body of evidence reinforces that access to immunisation is shaped not only by the physical presence of services but also by the time, financial and logistical burdens required to utilise them. As such, services that are technically available may remain effectively inaccessible when distance, indirect costs and prolonged waiting times are high, limiting equitable uptake even in settings where immunisation programmes are available. The findings on quality of care suggest that interpersonal experience and communication were central to how caregivers judged immunisation services. Quality composite scores were generally high in several LGAs, especially Guyuk, Gombi, Mayo-Belwa and Ganye, reflecting favourable ratings on respectful treatment, provider competence, communication and service organisation. However, important gaps remained. Toungo had the lowest quality score, while Fufore stood out for higher reports of rude or disrespectful treatment. The qualitative findings are consistent with these patterns. In some LGAs, caregivers described health workers as respectful, calm, competent and helpful in explaining vaccines, side effects and next visit dates. In others, they described poor attention, rude behaviour, inadequate explanation and weak queue management. These findings align with evidence from other settings showing that the quality of immunisation services is shaped not only by technical delivery but also by whether caregivers feel respected, informed and treated fairly throughout the visit. In urban communities in Sierra Leone, Jalloh et al.^40^ found that positive experiences with health workers acted as practical enablers of childhood vaccination, whereas feeling disrespected by health workers and expecting to pay money for services that should be free were important barriers. Caregivers in that study also expressed a desire for more convenient and positive clinic experiences. Similarly, findings from First Nations communities in Canada showed that some families felt dismissed or disrespected during clinic encounters, which contributed to mistrust of the health system and reduced adherence to recommended immunisation schedules. The same study also highlighted that strong relationships with healthcare providers, culturally respectful care and effective communication were important enablers of childhood immunisation.^41^ Taken together, these studies reinforce our findings that respectful treatment, clear communication and fair service processes are critical dimensions of immunisation service quality and strongly influence caregiver trust, satisfaction and continued use of services. Despite weaknesses in immunisation service delivery, satisfaction with routine immunisation services remained high in most LGAs, and willingness to return for the next immunisation visit was consistently strong. Quantitative findings showed that satisfaction was highest in Guyuk and remained strong in Ganye, Mubi North, Maiha and Numan. Qualitative findings showed that caregivers often linked satisfaction to the perceived health benefits of vaccination, positive staff interactions and confidence that immunisation protects children from disease. Even in settings where caregivers described delays, crowding or poor staff attitude, many still stated that they would return because they believed the service was important for their children’s health. This pattern is comparable to findings from Addis Ababa, Ethiopia and Cameroon, where most caregivers reported satisfaction with immunisation services, yet important concerns persisted around long waiting times, being turned away without receiving services and inadequate information about outreach activities.^39,42^ The positive overall satisfaction or continued willingness to return from our study should not be interpreted as evidence that service-related bottlenecks are inconsequential. Rather, caregivers may continue using services because they strongly value the protective benefits of immunisation even while absorbing the burden of delays, crowding, poor staff attitude and other avoidable barriers. Over time, however, such negative experiences may erode confidence, increase the opportunity cost of attendance and threaten continuity of uptake if they are not addressed.

### Strengths and limitations of study

This study has several strengths. The mixed-methods design enhanced the validity and depth of findings through the integration of quantitative and qualitative data. The relatively large sample size and the use of probability proportional to size sampling improved representativeness. In addition, the use of Community Score Card enabled a comprehensive assessment of immunisation service delivery. However, this study also has some limitations. Caregiver responses were self-reported and may be subject to recall and social desirability bias. Furthermore, the study was conducted in selected STRICAAN-supported LGAs and facilities in Adamawa State, which may limit the generalisability of the findings to non-supported areas.

### Recommendation and Implication for practice

Poorer-performing LGAs, in our study, appear to face overlapping operational and experiential weaknesses, including delays, long waiting times, weaker communication, poorer interpersonal care and less reliable service organisation. These patterns point to the need for targeted improvement strategies rather than a one-size-fits-all response. Strengthening session timeliness, reducing waiting time, improving health worker availability, ensuring consistent vaccine supply and reinforcing respectful communication may yield meaningful gains, particularly in lower-performing LGAs. In addition, addressing localised operational gaps, especially in Toungo and Fufore, should be central to targeted improvement strategies.

## Conclusion

Routine immunisation services in Adamawa State are highly valued by caregivers, but their experiences vary across locations. Our findings show that strong service performance depends not only on availability but also on timeliness, affordability, respectful care, clear communication and responsiveness to caregiver needs. Although willingness to return remains high, this appears to be driven more by caregivers’ strong belief in the benefits of immunisation than by consistently positive service experiences. Many caregivers continue to use immunisation services despite delays, crowding and poor staff interactions, effectively bearing these challenges. However, if these immunisation service-delivery gaps persist, they may gradually undermine confidence, increase the cost and burden of accessing care and threaten sustained uptake over time.
